# The colorectal cancer conundrum: the rising burden in younger adults

**DOI:** 10.1016/j.ebiom.2025.105757

**Published:** 2025-05-14

**Authors:** eBioMedicine

In January, 2025, the American Cancer Society released their annual report on cancer statistics. Incidence of cancer has been rising for years, and the 2025 report noted that incidence continues to increase for six of the top ten cancers, namely breast, prostate, melanoma, uterine, pancreatic, and colorectal cancer. However, the report also noted a shift in the burden of cancer incidence, from older adults to younger adults, and from men to women.

For colorectal cancer in particular, incidence rates among older adults have dropped by about 1% per year between 2012 and 2021, but rates have actually *increased* by 2.4% per year in the same period in those younger than 50 years. Furthermore, the increase in colorectal incidence does not appear to be limited to the USA, but rather is a global phenomenon, according to a study in *The Lancet Oncology*. The study showed that colorectal cancer incidence among patients younger than 50 years is increasing in over 50 countries, from Japan to Denmark to Uganda, despite these countries having vastly different health-care systems and screening guidelines. What is behind this rather disturbing trend?

Increased cancer incidence can in some cases be a reflection of changes in diagnostic screening methods and policies. For example, reporting of prostate cancer incidence increased substantially with the introduction of prostate-specific antigen screening protocols and then decreased again from 2007 to 2014 with changes in screening recommendations. However, updates to screening guidelines for colorectal cancer in the USA were prompted by rising colorectal cancer incidence in younger adults: the recommended age of colorectal cancer screening changed from 50 years and older to 45 years and older in 2021. This change suggests that earlier screening might not be a central factor in this increasing incidence.

An alternate suggestion for the increased colorectal cancer incidence is the concomitant rise in obesity. The association between obesity and the risk of developing colorectal cancer, along with other malignancies such as thyroid and renal cancer, has been established. The underlying mechanisms are under investigation but have been suggested to involve persistent low-grade inflammation resulting from growth of fat cells, which might facilitate tumour growth. However, obesity is often also associated with insulin resistance, which is also linked to colorectal cancer risk; disentangling the two might prove to be challenging.

A further avenue of research is the role of the microbiome in colorectal tumour genesis. Several studies have shown increases of specific genotoxic gut bacteria in colorectal cancer tumours relative to healthy tissue, including polyketide sythetase positive (pks+) *Escherichia coli*, Enterotoxigenic *Bacteroides fragilis* and *Fusobacterium nucleatum*. One study found that the presence of pks+ *E coli* was associated with a particular somatic mutation in the adenomatous polyposis coli gene, whereas the presence of increased *F nuceleatum* was associated with DNA mismatch repair deficiency, BRAF V600E mutation, and proximal tumour location. A paper in *eBioMedicine* investigated the role of the gut microbiome in patients with Lynch syndrome, a hereditary condition that causes an increased risk of colorectal cancer throughout a patient's life. The authors found that both pks+ and pks– *E coli* and *F nucleatum* were enriched in the colorectal cancer tumours of patients with Lynch syndrome. Further investigation into the particulars of the microbiome's role in colorectal cancer could lead to the identification of alternate markers of risk and disease progression, which would be crucial tools if the burden of this cancer continues to grow.

Given that the increase in colorectal cancer incidence in younger adults is occurring globally, environmental factors have been suggested as a driver. One provocative idea exploring an environmental link is the role of microplastics. Microplastics are ubiquitous, and exposure to them begins at an early age. Moreover, the presence of microplastics within the human digestive tract have been confirmed in colectomy samples. A recent study in mice showed that microplastics at environmental doses disrupted a number of normal gastric functions, including mucus and gastric juice secretion, and destroyed normal mucosal barrier formation, all of which provide a favourable environment for tumourigenesis. In vitro, the same study showed that microplastics induced excessive reactive oxygen species, which are known to cause DNA damage linked to carcinogenesis. This topic is an area of active research, and further work is required to determine the relevance of these results to human health.

Irrespective of the mechanism for increased incidence, improved diagnostics could improve outcomes for all patients. Given that younger patients are not usually suspected to have colorectal cancer, tumours are usually found at a later stage, which makes treatment more challenging. Rapid, blood-based screening could assist in improving diagnostics in younger cohorts. Current screening programmes tend to use the faecal immunochemical test (FIT) for patients with average risk and then follow-up with a colonoscopy if results are positive. As such, the test has a high specificity of approximately 95%, meaning that a negative test will very likely be negative even with a colonoscopy; however, the sensitivity is less ideal, at around 70%. This sensitivity translates into a greater percentage of unnecessary follow-up colonoscopies due to false positive tests. Results of a cell-free blood-based assay and a multi-target stool test were published last year. Compared with the FIT test, both had slightly lower specificities (89.6% for the blood-based assay and 90.6% for the multi-target stool test), but had higher sensitivities (83.1% for the blood-based assay and 93.4% for the multi-target stool test). Both tests have been recently approved by the US Food and Drug Administration and now offer further options for first-line testing. Further improvements, particularly for blood-based tests, could assist in expanding screening and adherence to screening for this challenging disease.

Collectively, understanding the increased incidence of colorectal cancer in younger adults has multiple facets and avenues for investigation. In the meantime, improvements in diagnostics might allow both more rapid treatment and expansion of screening to catch these tumours at an earlier, more treatable stage. At *eBioMedicine*, we encourage studies aiming to understand both the complexities of this trend and proposed improvements to current screening methods for this, and all other tumour types.

